# Severe Hypercalcemia and Acute Kidney Injury in a Patient with Probable IgG4-Related Disease and Cholestatic Liver Injury: A Case Report

**DOI:** 10.3390/jcm15197498

**Published:** 2026-09-26

**Authors:** Chi-Hsien Chang, Shang-Feng Tsai

**Affiliations:** 1 Division of Gastroenterology, Department of Internal Medicine, Show Chwan Memorial Hospital, Changhua 500, Taiwan; 2 Department of Post-Baccalaureate Medicine, College of Medicine, National Chung Hsing University, Taichung 402, Taiwan; 3 Division of Nephrology, Department of Internal Medicine, Taichung Veterans General Hospital, 160, Sec. 3, Taiwan Boulevard, Taichung 407, Taiwan; 4 Division of Clinical Informatics, Department of Digital Medicine, Taichung Veterans General Hospital, Taichung 407, Taiwan

**Keywords:** IgG4-related disease (IgG4-RD), hypercalcemia, acute kidney injury (AKI), possible IgG4-related sclerosing cholangitis (IgG4-SC), suspected immune thrombocytopenia (ITP)

## Abstract

We report a 54-year-old man with a history of urticaria, hyperlipidemia, and hypertension who was receiving regular bisoprolol, rosuvastatin, and verapamil. He initially developed severe hypercalcemia (18.56 mg/dL) and acute kidney injury (serum creatinine, 5.14 mg/dL) while in China. Following intravenous hydration and calcitonin, serum calcium normalized and renal function substantially improved. During hospitalization, he subsequently developed marked eosinophilia, severe thrombocytopenia, cholestatic liver injury, lymphadenopathy, and markedly elevated serum IgG4 (851 mg/dL). Extensive evaluation did not identify evidence of malignancy or infection. The overall findings raised clinical suspicion of IgG4-related disease (IgG4-RD) with possible hepatobiliary involvement and suspected immune-mediated thrombocytopenia. Ursodeoxycholic acid and fenofibrate were initiated when primary biliary cholangitis remained under consideration, followed by systemic corticosteroid therapy with dexamethasone. The patient’s liver abnormalities, thrombocytopenia, eosinophilia, and systemic manifestations subsequently improved. However, definitive tissue confirmation of IgG4-RD was not obtained, and characteristic cholangiographic evidence of IgG4-related sclerosing cholangitis was unavailable because MRCP or ERCP was not performed. During six months of outpatient follow-up, renal function remained stable and no clinical or biochemical relapse was observed. This case highlights severe hypercalcemia and acute kidney injury as unusual presenting features in a patient with clinical suspicion of IgG4-RD and emphasizes the importance of cautious diagnostic interpretation when histopathological and cholangiographic confirmation is unavailable.

## 1. Introduction

IgG4-related disease (IgG4-RD) is a systemic immune-mediated fibroinflammatory disorder characterized by tumefactive organ enlargement, elevated serum IgG4 concentrations, and dense lymphoplasmacytic infiltration rich in IgG4-positive plasma cells. The disease can affect nearly any organ system, with the pancreas, biliary tract, salivary glands, kidneys, and retroperitoneum being the most commonly involved sites. Because of its diverse clinical manifestations and ability to mimic malignancy, infection, or other autoimmune diseases, IgG4-RD often presents a significant diagnostic challenge. In this report, we describe a patient who initially presented with severe hypercalcemia and acute kidney injury (AKI) and subsequently developed cholestatic liver injury, marked eosinophilia, thrombocytopenia, and lymphadenopathy in the setting of markedly elevated serum IgG4. The coexistence and temporal evolution of these multisystem manifestations posed a diagnostic challenge and raised suspicion of IgG4-RD. This case is notable for the combination of severe hypercalcemia and AKI as the initial presentation followed by hepatobiliary and hematological abnormalities, highlighting the need for comprehensive evaluation of alternative diagnoses and cautious interpretation of serum IgG4 elevation.

## 2. Case Report

A 54-year-old man with a history of urticaria, hyperlipidemia, and hypertension was under regular follow-up at a cardiovascular outpatient clinic. His regular medications included bisoprolol, rosuvastatin, and verapamil. Four days before admission to our hospital, while in China, laboratory testing at a local hospital showed severe hypercalcemia (serum calcium, 18.56 mg/dL) and AKI (SCr, 5.14 mg/dL). He received emergency treatment with intravenous hydration and calcitonin, resulting in partial improvement. After returning to Taiwan, he was admitted for further evaluation. On admission, laboratory tests showed normalized serum calcium (9.5 mg/dL), while SCr had decreased to 3.54 mg/dL. After tracing his history, he also had diarrhea, malaise, and pruritus. The patient was treated with intravenous hydration, with loop diuretics administered when edema developed. Renal function initially worsened, with SCr increasing from 3.54 to 4.42 mg/dL, followed by progressive improvement to 2.24, 1.51, and 1.25 mg/dL (Figure 1A). Renal function subsequently improved substantially, with SCr declining to 1.25 mg/dL by hospital day 7 and further to 0.67 mg/dL at discharge without recurrence of hypercalcemia. The urinary calcium-to-creatinine ratio (UCa/UCr) was approximately 2.0 mg/mg, which was above the expected range for normal urinary calcium excretion, consistent with marked urinary calcium excretion. Given the severity of the initial hypercalcemia, an extensive evaluation was undertaken. Laboratory testing revealed an intact parathyroid hormone level of 93.5 pg/mL (reference range: 15–65 pg/mL) in the setting of a total calcium level of 8.5 mg/dL, along with reduced 25-hydroxyvitamin D levels (25.1 ng/mL). This value was obtained after normalization of serum calcium and therefore does not represent the PTH level during the initial hypercalcemic episode. PTHrP and serum 1,25-dihydroxyvitamin D were not measured during the episode of severe hypercalcemia. Thyroid and parathyroid ultrasonography demonstrated mild enlargement of the right parathyroid gland (approximately 6 mm) and multiple enlarged lymph nodes in the bilateral neck regions measuring up to 27 mm. Serum and urine protein electrophoresis (SPEP/UPEP), immunofixation electrophoresis, and serum free light-chain assays were also performed, with no evidence of a monoclonal gammopathy or plasma cell dyscrasia. Evaluation for malignancy-associated hypercalcemia showed negative tumor markers, including AFP (<2 ng/mL), CEA (3.48 ng/mL), and CA19-9 (17.34 U/mL), although squamous cell carcinoma antigen (8.2 ng/mL) was elevated. Chest radiography revealed no active pulmonary lesions. Further evaluation with ear–nose–throat endoscopy showed no evidence of malignancy. Both upper and lower gastrointestinal endoscopic examinations were unremarkable. Neck computed tomography (CT) revealed several small lymph nodes in the bilateral neck regions. Whole-body bone scan and long-bone radiographs did not demonstrate bone metastases or osteolytic lesions. Abdominal computed tomography revealed multiple small para-aortic lymph nodes with enlarged bilateral kidneys (14.6 and 14.8 cm) without splenomegaly or other evidence of portal hypertension. An otolaryngology consultation was obtained for excisional biopsy of the cervical lymph nodes (but only fine-needle biopsy was performed due to the high risk of bleeding). Positron emission tomography showed no evidence of malignancy. Although malignancy cannot be completely excluded without long-term follow-up, the overall clinical course strongly favored an inflammatory rather than neoplastic process. Albumin, ionized calcium, phosphate, magnesium, and acid–base parameters during the initial hypercalcemic episode were not available in the retrieved medical records. A reliable pre-illness baseline SCr/eGFR was also unavailable; therefore, complete recovery to a documented pre-illness renal baseline could not be established.

Eleven days after admission, the patient developed thrombocytopenia (platelet count 116 × 10^3^/μL) and marked eosinophilia (37.1%). By hospital day 14, prior to the scheduled lymph node biopsy, he experienced sudden worsening thrombocytopenia (platelet count 38 × 10^3^/μL) accompanied by persistent eosinophilia (30%). Disseminated intravascular coagulation studies showed a prothrombin time of 14.4 s, activated partial thromboplastin time of 34.5 s, fibrin degradation products of 3.8 μg/mL, fibrinogen of 409.1 mg/dL, and D-dimer of 1.39 mg/L. Because of concomitant thrombocytopenia and lymphadenopathy, hematologic malignancy was suspected. A bone marrow biopsy was therefore performed but revealed mild hypercellular marrow with increased eosinophils. Given the recent travel history, infectious and parasitic causes were considered; however, a comprehensive parasitological evaluation was not documented in the available records. Drug-related hypersensitivity was considered in the differential diagnosis; however, no specific drug challenge or formal drug-induced hypersensitivity assessment was performed. Platelet transfusion was administered; however, the platelet count continued to decline, reaching 6 × 10^3^/μL by hospital day 20. During the same period, the patient developed direct hyperbilirubinemia (direct bilirubin 1.69 mg/dL, total bilirubin 2.25 mg/dL) and hepatitis (AST 179 U/L, ALT 87 U/L), accompanied by elevated lactate dehydrogenase of 383 U/L and markedly increased alkaline phosphatase up to 778 U/L. He also experienced pruritus and fatigue. Abdominal CT showed no evidence of obstructive biliary disease or pancreatitis, and the patient did not report abdominal pain. There was no evidence of hemolysis, and haptoglobin levels remained within the normal range (2 g/L). Soluble interleukin-2 receptor levels were markedly elevated (>5000 pg/mL), along with increased ferritin levels (1446.32 ng/mL). Viral studies were negative, including hepatitis B virus, Epstein–Barr virus, cytomegalovirus, and herpes simplex virus. Autoimmune testing using an immunofluorescence assay showed negative antinuclear antibody (ANA; titer 1:80) and antineutrophil cytoplasmic antibody (ANCA) results; however, a cytoplasmic reticular/anti-mitochondrial antibody pattern (AC-21) on indirect immunofluorescence (IIF) was detected, with an anti-mitochondrial antibody (AMA) titer of 1:320. Anti–smooth muscle antibodies (ASMAs) were positive (titer 1:40), whereas AMA was negative by enzyme-linked immunosorbent assay (ELISA). Primary biliary cholangitis (PBC) with autoimmune hepatitis (AIH) overlap syndrome cannot be excluded. Immunologic testing also demonstrated markedly elevated serum IgG4 levels (851 mg/dL) (ratio of IgG4/total IgG=30.8%). Considering the markedly elevated serum IgG4 level, possible hepatobiliary involvement, systemic lymphadenopathy, bilateral kidney enlargement, and exclusion of major alternative diagnoses, clinically suspected IgG4-RD was considered the most likely unifying clinical impression. Because PBC could not be excluded, ursodeoxycholic acid (900 mg/day) and fenofibrate (160 mg/day) were initiated on hospital day 18. Because of the persistent multisystem inflammatory manifestations and markedly elevated serum IgG4, dexamethasone (10 mg/day) was subsequently initiated on hospital day 21 for the suspected immune-mediated inflammatory process. Three days after the initiation of treatment, hepatic function and jaundice began to improve, with total bilirubin decreasing from 3.77 to 3.16 mg/dL, alkaline phosphatase declining from 890 to 771 U/L, and AST decreasing from 222 to 196 U/L. The platelet counts also began to recover, increasing from 9 to 27 × 10^3^/μL. Eosinophilia also improved, decreasing from 64.9% to 2%. A sonography-guided neck lymph node biopsy was performed on day 27 after admission; however, the pathology revealed no evidence of lymphoma and the number of IgG4-positive plasma cells did not meet the diagnostic threshold for IgG4-RD, although the biopsy was performed after approximately one week of high-dose corticosteroid therapy. On day 29 after admission, he was discharged with normalized calcium levels (7.9 mg/dL; peak 18.56 mg/dL) and renal function (serum creatinine 0.67 mg/dL; peak 5.14 mg/dL), along with improvement in jaundice (total bilirubin 2.65 mg/dL; peak 3.77 mg/dL), hepatitis (AST 117 U/L; peak 222 U/L) (Figure 1B), and thrombocytopenia (platelet count 165 × 10^3^/µL; nadir 6 × 10^3^/µL) (Figure 1C). The final clinical impression was clinically suspected IgG4-RD with possible hepatobiliary involvement and possible IgG4-related sclerosing cholangitis, with suspected immune-mediated thrombocytopenia; PBC and AIH were considered alternative diagnoses. The major clinical events, diagnostic assessments, therapeutic interventions, and follow-up are summarized in Table 1. The patient was followed in the outpatient clinic for 6 months after discharge. Prednisolone was gradually tapered to 5 mg every other day, with no evidence of clinical or biochemical relapse. Serial laboratory data during hospitalization are presented in Figure 1, whereas post-discharge follow-up data are presented in Figure 2.

## 3. Discussion

The diagnosis of IgG4-RD in this patient deserves careful consideration. Histopathological confirmation remains the gold standard for diagnosis and is incorporated into both the 2020 Revised Comprehensive Diagnostic (RCD) Criteria [1] and the 2019 ACR/EULAR Classification Criteria [2]. However, the latter were primarily developed for research classification rather than clinical diagnosis. In the present case, histopathological confirmation was unavailable because no appropriate target organ was accessible for biopsy. Cervical lymph node biopsy was performed only after approximately one week of high-dose corticosteroid therapy and demonstrated no evidence of lymphoma, while IgG4-positive plasma cell infiltration did not meet the diagnostic threshold. It is well recognized that lymph nodes are not the preferred biopsy site for IgG4-RD because they frequently lack the characteristic histopathological features of storiform fibrosis and obliterative phlebitis, and corticosteroid treatment before biopsy may further reduce IgG4-positive plasma cell infiltration. Assessment according to the 2020 RCD criteria further illustrates the diagnostic uncertainty in this case. The patient fulfilled the clinical/radiological domain, with bilateral kidney enlargement (possible renal involvement) and multisystem involvement, and the serological domain, with a serum IgG4 level of 851 mg/dL. However, the pathological domain was not fulfilled because the cervical lymph-node biopsy, performed after approximately one week of high-dose corticosteroid therapy, did not demonstrate sufficient IgG4-positive plasma-cell infiltration or characteristic fibrosis. Accordingly, the patient fulfills the possible IgG4-RD category according to the 2020 RCD criteria, rather than definite IgG4-RD. Despite the absence of definitive histopathological confirmation, the markedly elevated serum IgG4 level, multisystem clinical manifestations, and overall clinical course supported clinically suspected IgG4-RD as the most likely unifying clinical impression. The serum IgG4 level was 851 mg/dL, with an IgG4/total IgG ratio of 30.8%, substantially exceeding the previously reported optimal diagnostic cutoff of 11.4% (sensitivity, 96.2%; specificity, 92.1%) [3]. However, an elevated serum IgG4 level is not specific for IgG4-RD, and the observed organ manifestations could not be definitively attributed to IgG4-RD. The coordinated clinical and biochemical improvement following corticosteroid therapy provided supportive evidence of an immune-mediated inflammatory process but was not disease-specific. Taken together, these findings support clinically suspected IgG4-RD as the most likely unifying clinical impression, while the absence of definitive histopathological confirmation remains an important limitation.

One notable feature of the present case is the occurrence of severe hypercalcemia and AKI as the initial presentations. Severe hypercalcemia has been reported in association with IgG4-RD, with only a small number of cases reported in the literature [4,5,6,7,8]. The underlying mechanism of hypercalcemia reported in association with IgG4-RD remains incompletely understood, and several hypotheses have been proposed. First, the inflammatory milieu in IgG4-RD may resemble that observed in granulomatous diseases and promote extrarenal production of active vitamin D [5]. In IgG4-RD, macrophages are known to shift toward a pro-fibrotic M2-like phenotype [9,10,11,12] and may overexpress innate immune receptors [10]. These activated macrophages can express 1α-hydroxylase, which enhances the conversion of 25-hydroxyvitamin D to its active form, 1,25-dihydroxyvitamin D. This process increases intestinal calcium absorption and may ultimately lead to hypercalcemia [13,14]. In addition, PTH-dependent hypercalcemia has also been reported in association with IgG4-RD [5,15]. Finally, autoantibodies targeting the calcium-sensing receptor have been proposed as another potential mechanism [15]. In the present patient, the relatively low 25-hydroxyvitamin D level (25.1 ng/mL) is compatible with, but does not establish, increased conversion to its active metabolite. Because serum 1,25-dihydroxyvitamin D was not measured during the hypercalcemic episode, a calcitriol-mediated mechanism cannot be confirmed in this patient. Thus, the proposed macrophage-mediated extrarenal 1α-hydroxylase mechanism should be regarded as a hypothesis based on previously reported mechanisms rather than a demonstrated mechanism in the present case. Unlike granulomatous disorders such as sarcoidosis, IgG4-RD is characterized by lymphoplasmacytic infiltration and storiform fibrosis rather than macrophage-rich granulomatous inflammation, which may partly explain why dysregulated extrarenal 1,25-dihydroxyvitamin D production and hypercalcemia have only rarely been reported in association with IgG4-RD.

An intriguing serological finding in this patient was the presence of an anti-cytoplasmic reticular pattern on IIF with a titer of 1:320, while ELISA testing for AMA was negative. The AC-21 cytoplasmic reticular pattern observed on HEp-2 cell-based ANA testing is classically associated with AMA and is most frequently reported in patients with PBC [16,17]. However, IIF primarily identifies morphological staining patterns rather than specific mitochondrial antigens. In contrast, ELISA-based assays detect antibodies directed against defined mitochondrial epitopes, most commonly the AMA-M2 subtype, which represents the most characteristic serologic marker of PBC [18]. Several mechanisms may explain the discordance between IIF and ELISA observed in this case. First, IIF may detect AMA subtypes other than M2 (such as M1, M3, M5, or M8), which are not routinely captured by standard ELISAs targeting AMA-M2 [19]. Second, IIF is generally more sensitive but less specific than antigen-specific immunoassays, and low-affinity or low-titer antibodies may produce cytoplasmic reticular staining while remaining below the detection threshold of ELISA. Third, non-mitochondrial autoantibodies may occasionally generate similar cytoplasmic reticular fluorescence patterns on HEp-2 cells, potentially mimicking AMA-related staining. Clinically, the patient presented with cholestatic liver dysfunction accompanied by pruritus and fatigue, features commonly observed in immune-mediated cholangiopathies. Because PBC was initially suspected, ursodeoxycholic acid was initiated as standard therapy, with fenofibrate added as adjunctive therapy for cholestatic biochemical abnormalities. However, corticosteroid therapy was subsequently introduced for the clinically suspected systemic IgG4-RD-related inflammatory process. Notably, the patient demonstrated rapid improvement in cholestatic liver enzymes and jaundice after the initiation of high-dose corticosteroid therapy. This prompt steroid response was compatible with a steroid-responsive inflammatory hepatobiliary process but was not specific for IgG4-RD. Given the absence of characteristic biliary imaging and liver histopathology, IgG4-related sclerosing cholangitis (IgG4-SC) could not be definitively established.

However, some reported cases have suggested a possible overlap between IgG4-RD and PBC, although the distinction between these conditions can be challenging because of overlapping clinical and serological features [20,21,22,23,24]. In one reported case [21], AMA-M2 positivity led the authors to propose possible coexistence of IgG4-SC and PBC. However, the available evidence in such cases should be interpreted cautiously, as AMA positivity alone does not definitively establish the diagnosis of PBC. In our patient, the rapid biochemical improvement following corticosteroid therapy was compatible with a steroid-responsive inflammatory hepatobiliary process, but was not specific for IgG4-SC, particularly because ursodeoxycholic acid and fenofibrate had been initiated before corticosteroid therapy. Overall, true coexistence of IgG4-RD and PBC appears to be uncommon. The two diseases are considered to have distinct immunopathogenic mechanisms: IgG4-RD is characterized by a Th2- and regulatory T cell-dominant fibroinflammatory process [25], whereas PBC is an autoimmune cholangitis predominantly associated with antimitochondrial antibodies targeting the small intrahepatic bile ducts [26,27]. These differences highlight the diagnostic challenges in distinguishing IgG4-related hepatobiliary involvement from PBC, particularly when characteristic imaging or histopathological findings are unavailable.

In summary, IgG4-related hepatobiliary involvement is clinically suspected based on the markedly elevated serum IgG4 level, cholestatic/hepatitic liver abnormalities, multisystem manifestations, and response to corticosteroid therapy; however, IgG4-SC cannot be established definitively in the absence of characteristic biliary imaging or histopathological confirmation. The presence of an AMA-like IIF pattern together with cholestatic liver dysfunction highlights the possibility of serologic overlap and illustrates the diagnostic complexity of autoimmune hepatobiliary disorders. These discordant serologic findings underscore the importance of integrating serological patterns with clinical features, imaging studies, and treatment response when evaluating patients with immune-mediated cholangiopathies.

In addition, this patient developed severe thrombocytopenia during hospitalization, which complicated the diagnostic evaluation by limiting the feasibility of further invasive procedures. Hematologic abnormalities in IgG4-RD are relatively uncommon but have been reported in the literature [28]. Proposed mechanisms include immune-mediated platelet destruction, hypersplenism secondary to hepatobiliary involvement, or broader systemic immune dysregulation. Eleven case reports have reported ITP occurring in association with IgG4-RD [28,29,30], raising the possibility that autoimmune mechanisms may contribute to platelet destruction in a subset of patients with clinically suspected IgG4-RD. However, this remains uncommon, as IgG4 is not the predominant IgG subclass typically implicated in ITP [31]. To date, only one study has specifically investigated this issue, demonstrating that the antiplatelet antibody [32] belonged to the IgG4 subclass. In this patient, the rapid improvement in platelet counts following corticosteroid therapy (from 6 × 10^3^/μL to 165 × 10^3^/μL) was compatible with an immune-mediated mechanism, although secondary ITP cannot be established definitively in the absence of platelet-specific antibody testing. Although the overall prevalence of hematologic manifestations in IgG4-RD remains uncertain, clinicians should be aware that such abnormalities may occur and may complicate both diagnostic evaluation and clinical management.

Overall, this case highlights several uncommon clinical findings occurring in association with clinically suspected IgG4-RD, including severe hypercalcemia, AKI, hepatobiliary abnormalities, and severe thrombocytopenia. These features may mimic malignancy, endocrine disorders, or other systemic diseases, leading to extensive investigations before a definitive diagnosis is established. Recognition of the multisystem nature of IgG4-RD is therefore essential, particularly in patients presenting with unexplained systemic inflammatory findings and elevated serum IgG4 levels. Importantly, IgG4-RD generally responds well to corticosteroid therapy, which remains the first-line treatment. Early recognition and timely initiation of immunosuppressive therapy may prevent progressive organ damage and improve clinical outcomes.

## 4. Conclusions

This case highlights severe hypercalcemia and acute kidney injury as unusual presenting features in a patient with probable IgG4-related disease and multisystem inflammatory manifestations. Although the markedly elevated serum IgG4 level, multisystem involvement, exclusion of major alternative diagnoses, and subsequent clinical improvement support a clinical suspicion of IgG4-RD, the absence of definitive histopathological confirmation limits diagnostic certainty. The mechanism underlying the severe hypercalcemia also remains uncertain, as PTH during hypercalcemia and serum 1,25-dihydroxyvitamin D were not measured. This case therefore illustrates both the diagnostic challenge and the need for cautious interpretation when severe hypercalcemia occurs in the setting of clinically suspected IgG4-RD.

## Figures and Tables

**Figure 1:**
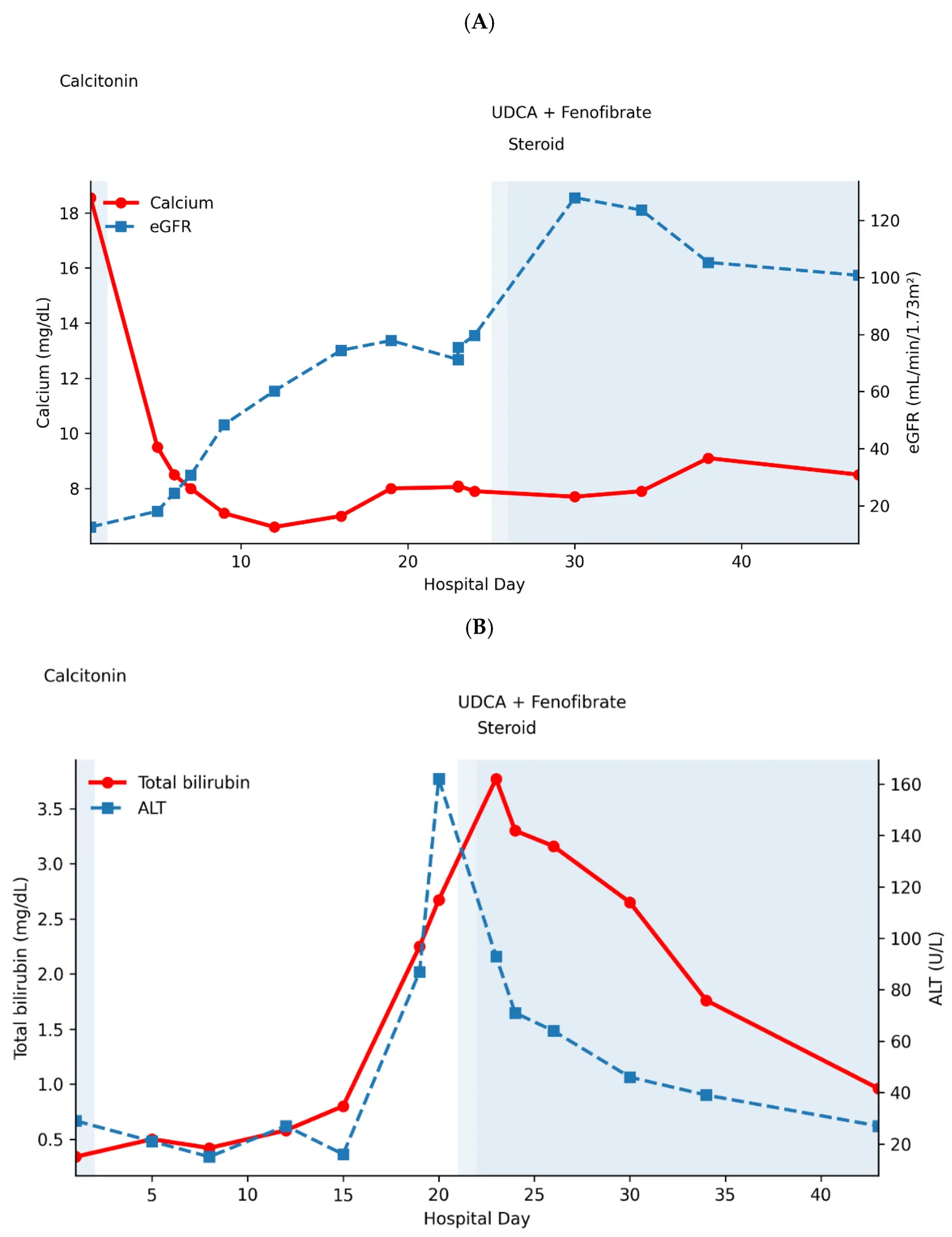

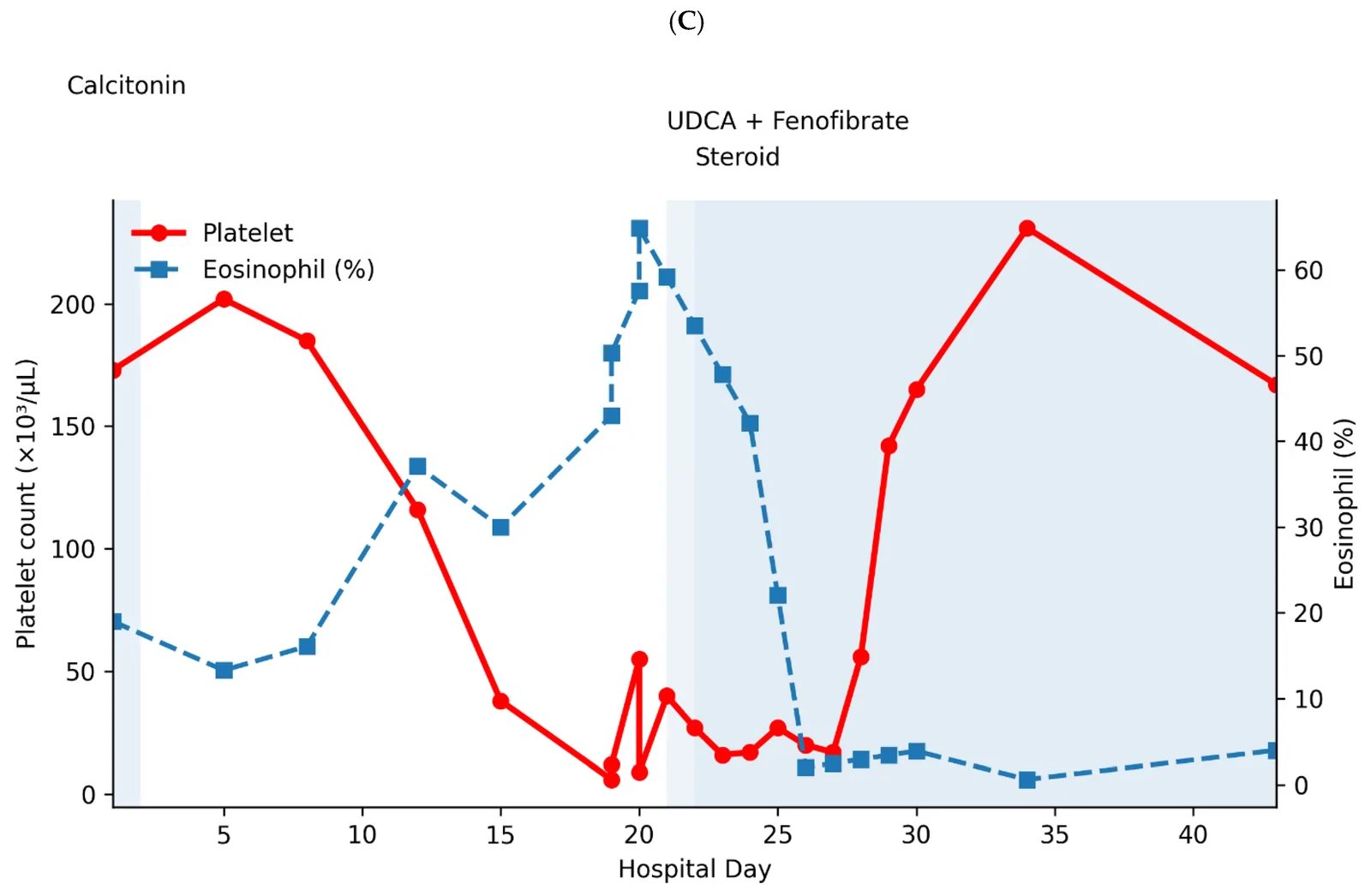
Multisystem clinical and laboratory changes during hospitalization. Observed laboratory measurements during hospitalization are presented without interpolation between time points. Treatment interventions are indicated according to their actual timing. Pre-admission values are identified separately from in-hospital measurements, and post-discharge follow-up data are presented separately in Figure 2. Missing laboratory measurements were not estimated. (**A**) Serum calcium (left y-axis) and estimated glomerular filtration rate (eGFR; right y-axis) demonstrate severe initial hypercalcemia and transient worsening of renal function, followed by progressive recovery of kidney function and normalization of serum calcium. (**B**) Total bilirubin (left y-axis) and alanine aminotransferase (ALT; right y-axis) show transient elevation followed by gradual recovery, indicating hepatic involvement. (**C**) Platelet count (left y-axis) and eosinophil percentage (right y-axis) reveal an inverse temporal relationship, with eosinophilia preceding platelet nadir and subsequent recovery after treatment. Calcitonin was administered during hospital days 1–2. Ursodeoxycholic acid and fenofibrate were initiated on hospital day 18, followed by corticosteroid therapy on day 21. Only actual laboratory measurements are plotted; lines connecting data points are provided solely to illustrate temporal trends. Absolute eosinophil counts were unavailable for all relevant time points.

**Figure 2:**
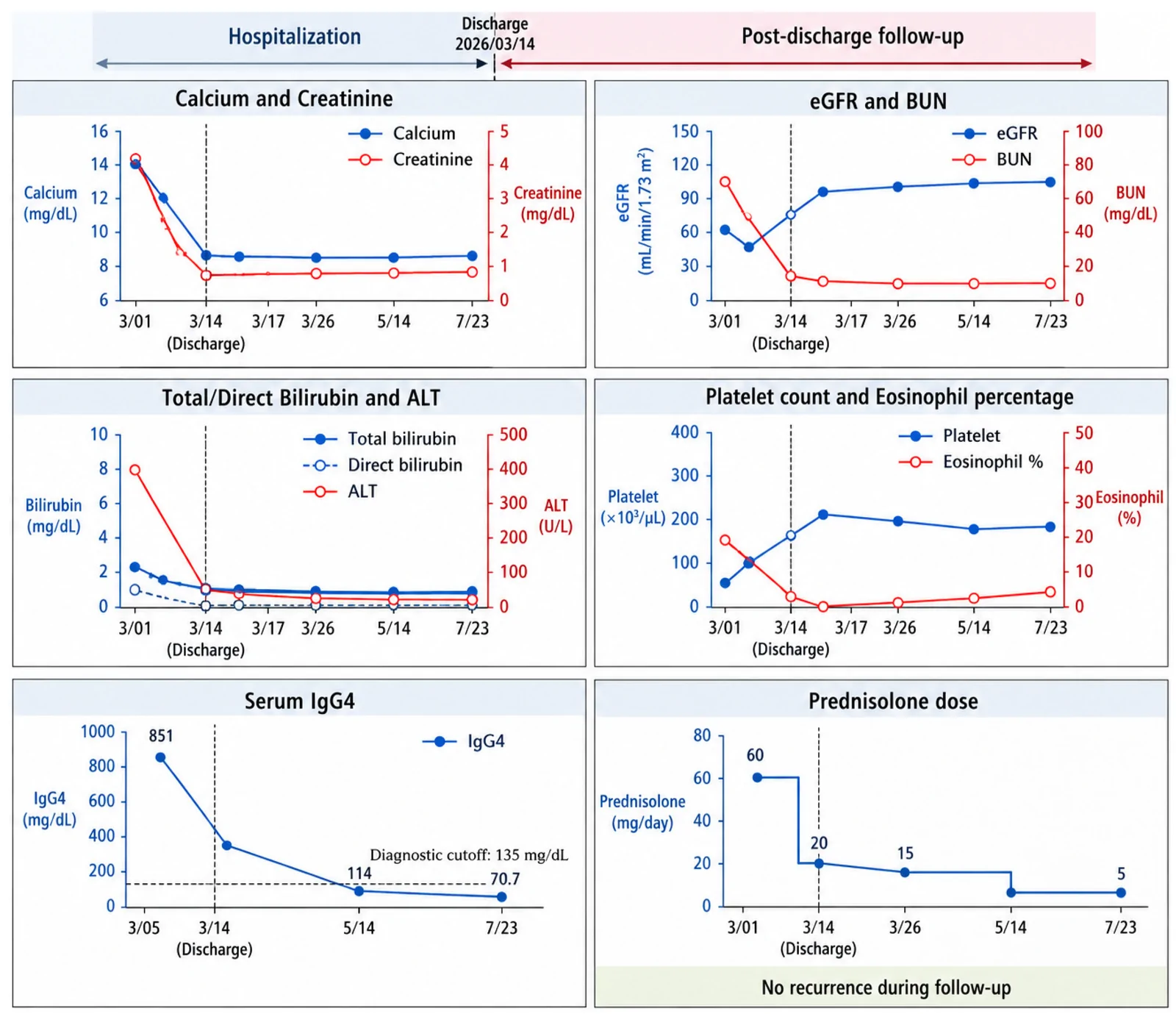
Clinical course and post-discharge follow-up. Serial changes in renal function, serum calcium, hepatobiliary parameters, hematologic abnormalities, and serum IgG4 levels during hospitalization and after discharge. The patient was discharged on 14 March 2026. Following discharge, renal function remained stable, with serum creatinine ranging from 0.77 to 0.93 mg/dL and eGFR remaining above 96 mL/min/1.73 m^2^. Serum bilirubin and ALT progressively improved, while platelet counts remained within the normal range and eosinophilia did not recur. Serum IgG4 decreased from 851 mg/dL during hospitalization to 114 mg/dL on May 14 and 70.7 mg/dL on July 23. No recurrence was documented during follow-up. Only actual laboratory measurements are shown; no values were interpolated.

**Table 1:** Timeline of major clinical events, diagnostic assessments, therapeutic interventions, and follow-up.

Time Point	Clinical Course/Key Findings	Diagnostic Assessment	Therapeutic Intervention/Outcome
Before admission (China)	Severe hypercalcemia (18.56 mg/dL) and AKI (SCr 5.14 mg/dL).	Initial laboratory assessment in China.	IV hydration and calcitonin; partial improvement.
Hospital day 1	Calcium normalized (9.5 mg/dL); SCr 3.54 mg/dL after return to Taiwan.	Initial inpatient evaluation for hypercalcemia and AKI.	IV hydration; loop diuretics when edema developed.
Hospital day 7	SCr improved to 1.25 mg/dL; no recurrent hypercalcemia.	Serial renal and calcium monitoring.	Supportive management continued.
Hospital days 11–14	Thrombocytopenia (116 → 38 × 10^3^/μL) with marked eosinophilia (37.1% → 30%).	DIC studies and hematologic evaluation; hematologic malignancy considered.	Platelet transfusion; bone marrow biopsy.
Hospital day 18	Cholestatic liver injury with jaundice, pruritus, and fatigue.	Autoimmune/hepatobiliary evaluation; PBC and AIH considered.	UDCA 900 mg/day + fenofibrate 160 mg/day initiated.
Hospital day 20	Platelet count nadir 6 × 10^3^/μL; eosinophilia persisted.	Continued evaluation for immune, infectious, and hematologic causes.	Supportive hematologic management.
Hospital day 21	Persistent multisystem inflammatory manifestations; serum IgG4 851 mg/dL.	Probable IgG4-RD considered.	Dexamethasone 10 mg/day initiated.
Hospital day 27	Liver tests, platelet count, and eosinophilia improved after corticosteroid therapy.	Cervical lymph-node excisional biopsy: no lymphoma; IgG4+ plasma cells below diagnostic threshold.	Corticosteroid therapy continued.
Hospital day 29 (discharge)	Ca 7.9 mg/dL; SCr 0.67 mg/dL; platelets 165 × 10^3^/μL; bilirubin and AST improving.	Probable IgG4-RD remained the clinical impression; definitive histopathology/cholangiography unavailable.	Discharged; subsequent prednisolone taper.
Post-discharge, 6-month follow-up	Stable renal function; no recurrent eosinophilia or thrombocytopenia; no clinical/biochemical relapse.	Serial outpatient renal, hepatic, hematologic, and IgG4 monitoring.	Prednisolone tapered to 5 mg every other day.

## Data Availability

All data relevant to this case report are included in the manuscript. No additional datasets are available.
